# Spatiotemporal modulations in heterotypic condensates of prion and α-synuclein control phase transitions and amyloid conversion

**DOI:** 10.1038/s41467-022-28797-5

**Published:** 2022-03-03

**Authors:** Aishwarya Agarwal, Lisha Arora, Sandeep K. Rai, Anamika Avni, Samrat Mukhopadhyay

**Affiliations:** 1 Centre for Protein Science, Design and Engineering, Indian Institute of Science Education and Research (IISER) Mohali, Punjab, India; 2 Department of Biological Sciences, Indian Institute of Science Education and Research (IISER) Mohali, Punjab, India; 3 Department of Chemical Sciences, Indian Institute of Science Education and Research (IISER) Mohali, Punjab, India

**Keywords:** Intrinsically disordered proteins, Protein aggregation

## Abstract

Biomolecular condensation via liquid-liquid phase separation of proteins and nucleic acids is associated with a range of critical cellular functions and neurodegenerative diseases. Here, we demonstrate that complex coacervation of the prion protein and α-synuclein within narrow stoichiometry results in the formation of highly dynamic, reversible, thermo-responsive liquid droplets via domain-specific electrostatic interactions between the positively-charged intrinsically disordered N-terminal segment of prion and the acidic C-terminal tail of α-synuclein. The addition of RNA to these coacervates yields multiphasic, vesicle-like, hollow condensates. Picosecond time-resolved measurements revealed the presence of transient electrostatic nanoclusters that are stable on the nanosecond timescale and can undergo breaking-and-making of interactions on slower timescales giving rise to a liquid-like behavior in the mesoscopic regime. The liquid-to-solid transition drives a rapid conversion of complex coacervates into heterotypic amyloids. Our results suggest that synergistic prion-α-synuclein interactions within condensates provide mechanistic underpinnings of their physiological role and overlapping neuropathological features.

## Abstract (web summary)

The authors show that prion protein and α-synuclein undergo phase separation through domain-specific electrostatic interactions. These complex coacervates possess electrostatic nanoclusters and can convert into multiphasic condensates and amyloids.

## Introduction

The precise spatiotemporal regulation of cellular machinery in its naturally crowded milieu is critical for the sustenance of life. Emerging evidence suggests a central role of liquid–liquid phase separation (LLPS) in maintaining subcellular organization via the formation of highly dynamic protein and nucleic-acid-rich biomolecular condensates, also known as membrane-less organelles^1–9^. Due to the absence of any delimiting membrane, these intracellular emulsions allow rapid exchange of components within the cellular environment and exhibit liquid-like behavior. These assemblies are highly context-dependent and facilitate an array of complex cellular functions ranging from chromatin reorganization to transcriptional regulation^1^. Given the multicomponent nature and complex functions associated with these condensates, they display different internal architectures^10–13^. For instance, nucleoli display distinct nested sub-compartments and hierarchal organization^14^. The components inside these condensates have been broadly classified into two categories namely, scaffolds and clients^15^. Scaffolds refer to the resident biomolecules with multiple interaction motifs which drive the formation of these multicomponent condensates via a dense network of intermolecular contacts involving electrostatic, hydrophobic, hydrogen bonding, dipole–dipole, π–π, and cation–π interactions^16–22^. On the other hand, biomolecules recruited via direct interactions with scaffolds, which are otherwise not required for the condensate formation are referred to as clients^15^. Intrinsically disordered proteins/regions (IDPs/IDRs) containing prion-like domains and low-complexity regions that can offer multivalent interaction sites can be considered as scaffolds governing the formation of these heterotypic condensates^23–28^. The physical origin of these condensates is dictated by the sequence architecture and composition of the scaffolds. These assemblies often comprise putative RNA-binding proteins such as Fused in Sarcoma (FUS) and FUS family proteins, which have been linked to various neurodegenerative diseases^29^. In some cases, these protein-rich liquid-like assemblies can potentially undergo deleterious liquid-to-solid transitions into ordered amyloid-like fibrils^29–35^.

In recent years, the emergence of overlapping neuropathological features has raised the possibility of heterologous aggregation of different amyloidogenic proteins^36^. In general, the co-existence of distinct pathologies is attributed to the synergistic interaction between different proteins, cross-seeding between unrelated proteins, or receptor-mediated toxicity^36–38^. For instance, a wealth of evidence suggests proximal locations or colocalizations of aggregates of unrelated amyloidogenic proteins such as α-synuclein (α-Syn), tau, amyloid-β in the brains of patients^39–41^. Along the same line, abnormal deposits of α-Syn in the form of Lewy bodies have been found in patients with sporadic or genetic prion diseases such as Creutzfeldt-Jakob disease (CJD), which is linked to the misfolding of the prion protein (PrP)^42^. Although PrP and α-Syn are independently known to aggregate and form cytoplasmic inclusions, their co-existence raises important questions about the underlying molecular mechanism. Recent studies have indicated that PrP acts as a receptor for α-syn oligomers and fibrils triggering the downstream signaling cascade resulting in cellular toxicity^43–46^. Also, inoculation of infectious prions in aged α-Syn transgenic mice has been associated with extensive α-Syn deposits^47^.

**Fig. 1:**
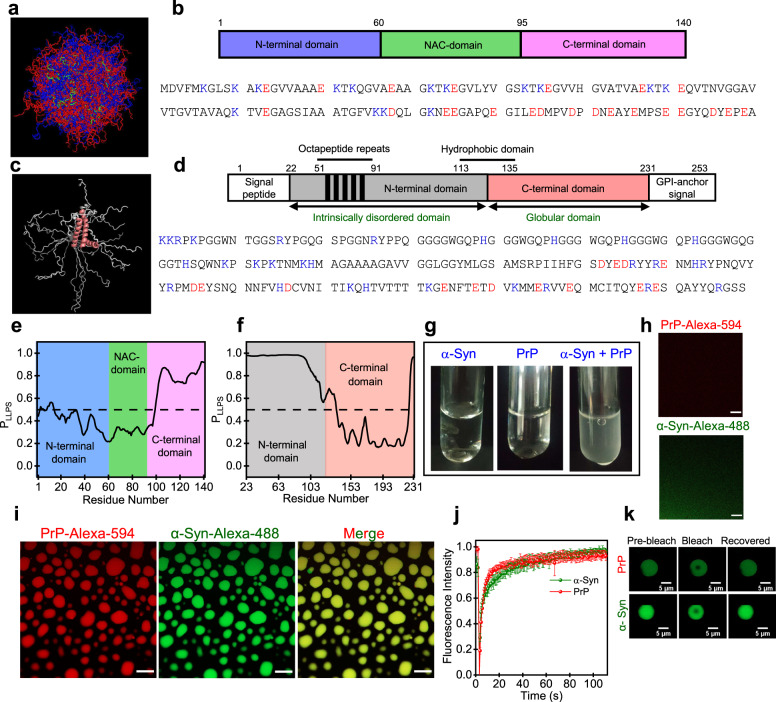
Heterotypic phase separation of α-Syn and PrP. **a** An overlay of 576 conformations obtained from the ensemble structure of α-Syn (PED ID: PED00024e001) generated using PyMOL (Schrödinger, LLC, New York). **b** Domain architecture and the amino acid sequence of α-Syn. Positively and negatively charged amino acids are shown in blue and red, respectively. **c** An overlay of 20 conformations obtained from the NMR structure of human PrP (90–231) (PDB ID: 2LSB) generated using PyMOL. **d** Schematic representation of PrP (23–231) indicating the N-terminal disordered and the C-terminal globular domains. Positively and negatively charged amino acids are shown in blue and red, respectively. Prediction of the LLPS propensity using FuzDrop for **e** α-Syn and **f** PrP (23–231). **g** LLPS upon mixing of homogenous solutions of α-Syn (45 µM) and PrP (30 µM) at pH 6.8 at 37 °C. **h**, **i** Confocal images of mixed homogeneous phases of PrP and α-Syn and complex coacervates of PrP (red) and α-Syn (green) performed using Alexa-594-labeled PrP (Cys 31) and Alexa-488-labeled α-Syn (Cys 90) indicating their complete colocalization (yellow) within droplets (Scale bar: 10 µm). See Supplementary Movie 1 for droplet fusion events. **j** FRAP kinetics of multiple droplets (~1% Alexa-488-labeled protein) for PrP (red) and α-Syn (olive). The FRAP experiments were performed using Alexa-488-labeled α-Syn and PrP independently. The data represent mean ± s.d for *n* = 5 independent experiments. Source data are provided as a Source Data file. **k** Fluorescence images of droplets during FRAP measurements. PrP and α-Syn concentrations were 20 and 30 µM, respectively. See Methods for details. The imaging was performed thrice with similar observations (**h**, **i**, **k**).

α-Syn is a 140-residue neuronal IDP, aggregation of which is associated with Parkinson’s disease. α-Syn contains three distinct regions namely, an amphipathic lysine-rich amino terminus (residues 1–60) with a highly conserved lipid-binding region, a central hydrophobic region known as the non-amyloid-β-component (NAC; residues 61–95) essential for aggregation, and an acidic carboxy-tail (residues 96–140) that interacts with metal ions and other proteins (Fig. 1a, b)^48^. Whereas, PrP is a 253-residue C-terminally glycophosphatidylinositol (GPI)-anchored protein consisting of two distinct regions namely, a highly flexible intrinsically disordered N-terminal segment (residues 23–120) and a globular C-terminal domain (residues 121–231) (Fig. 1c, d)^49,50^. The N-terminal IDR harbors several key regions such as the polybasic region comprising two lysine clusters (residues 23–30 and 100–110), a glycine-rich octapeptide repeat region (residues 51–90), and a hydrophobic segment (113–135). The globular C-terminal domain of PrP consists of three α-helices and two short antiparallel β-strands. The misfolding and aggregation of PrP has been associated with a class of invariably fatal and transmissible neurodegenerative diseases such as CJD^49^. In order to elucidate the molecular basis of their overlapping neuropathology, we set out to study the interaction between human α-Syn and PrP. In this work, we demonstrate that phase separation via a complex coacervation of α-Syn and PrP yields highly dynamic heterotypic condensates comprising ephemeral electrostatic nanoclusters within the liquid-like mesoscopic organization. Domain-specific interactions and charge anisotropies provide spatiotemporal modulations of these highly tunable and thermo-responsive condensates that eventually undergo maturation into highly ordered, heterotypic, solid-like amyloid fibrils.

## Results

### Heterotypic phase separation of α-Syn and PrP

In order to make predictions about the phase behavior of PrP and α-Syn, we first set out to evaluate the disorder and charge distribution in the primary amino acid sequence. As expected, disorder predictors revealed significant disorder for the entire sequence of the α-Syn and the N-terminal region of the PrP (Fig. S1a, b). The primary sequence of PrP and α-Syn carries a net positive (~+10) and negative charge (~−8), respectively, at a near-neutral pH. The linear net charge per residue (NCPR) plots generated using Classification of Intrinsically Disordered Ensemble Regions (CIDER)^51^ showed clustering of positive charges at the N-terminal part of PrP and negative charges preferentially located at the C-terminal part of α-Syn (Fig. S1c, d). We further evaluated the sequence-dependent phase behavior using well-known LLPS predictors FuzDrop^52^ and catGRANULE^53^, which revealed a significant phase separation propensity for both the proteins (Fig. 1e, f, Fig. S1e). It is interesting to note that the phase separation propensity is higher for the positively charged N-terminal segment of PrP as compared to its C-terminal domain that is well-folded. On the other hand, the phase separation propensity is higher for the negatively charged C-terminal domain of α-Syn as compared to its N-terminal and NAC domain. Therefore, we postulated that the electrostatic interactions could potentially promote their complex coacervation at the physiological pH. In order to experimentally verify if these two proteins together can undergo complex coacervation, we began by characterizing their phase behavior in vitro. Under our experimental condition, two separate solutions of PrP and α-Syn remained clear and dispersed at near-neutral pH (pH 6.8–7.4, 37 °C). To establish their monomeric nature, we also performed dynamic light scattering (DLS) measurements that revealed a hydrodynamic diameter of ~6 and ~10 nm for α-Syn and PrP, respectively, as expected for their monomeric hydrodynamic size (Fig. S1f, g). We next co-incubated PrP and α-Syn at different stoichiometries based on their net charges and observed their phase behavior using turbidity measurements and microscopic investigations. Upon the addition of PrP to α-Syn (molar ratio: α-Syn:PrP = 1.5, 37 °C), the solution spontaneously and rapidly turned turbid (Fig. 1g) indicating the presence of micron-sized condensates as also determined using DLS (Fig. S1h). Microscopic studies showed the presence of spherical liquid-like droplets that undergo fusion. The SDS-PAGE analysis also revealed the presence of both α-Syn and PrP in the sedimented condensed phase, establishing their heterotypic nature (Fig. S2a). Together, these experiments indicated the formation of heterotypic liquid-like complex coacervates of α-Syn and PrP.

In order to directly observe the presence of both α-Syn and PrP in these liquid droplets, we performed two-color confocal fluorescence imaging. We created single cysteine mutants at residues 90 and 31 of α-Syn and PrP, respectively, to perform site-specific fluorescence labeling using thiol-active fluorescent dyes namely, AlexaFluor-488 (green) and AlexaFluor-594 (red). PrP and α-Syn doped with their respective fluorescently labeled proteins (~1%) were mixed and observed using confocal microscopy. These images revealed the complete colocalization of PrP and α-Syn within these liquid droplets (Fig. 1h, i). The condensed phase concentration of PrP and α-Syn in these droplets was estimated to be ~10 and ~15 mM, respectively, compared to the dispersed phase concentration of ~15 and ~25 µM, respectively (Fig. S2b-d). We next examined the internal mobility of both these proteins within the condensates by utilizing fluorescence recovery after photobleaching (FRAP) kinetics. Both PrP and α-Syn revealed fast and complete recovery indicating their fast translational diffusion within these condensates (Fig. 1j, k). These droplets remained liquid-like even after ~5 h as also indicated by our confocal imaging and time-dependent FRAP measurements (Fig. S2e, f). Taken together, these studies highlight the highly dynamic nature of these heterotypic condensates in which both α-Syn and PrP exhibit complete miscibility and high molecular mobility within the mesoscopic condensed phase. We next hypothesized if these heterotypic condensates are formed due to electrostatic interactions between oppositely charged disordered domains of the two proteins similar to a complex coacervation of polyelectrolytes via charge neutralization that has been previously observed for other proteins and nucleic acids^54,55^. Therefore, we next set out to unmask the role of the electrostatic effects in heterotypic LLPS of α-Syn and PrP.

### Charge neutralization drives heterotypic LLPS of α-Syn and PrP

**Fig. 2:**
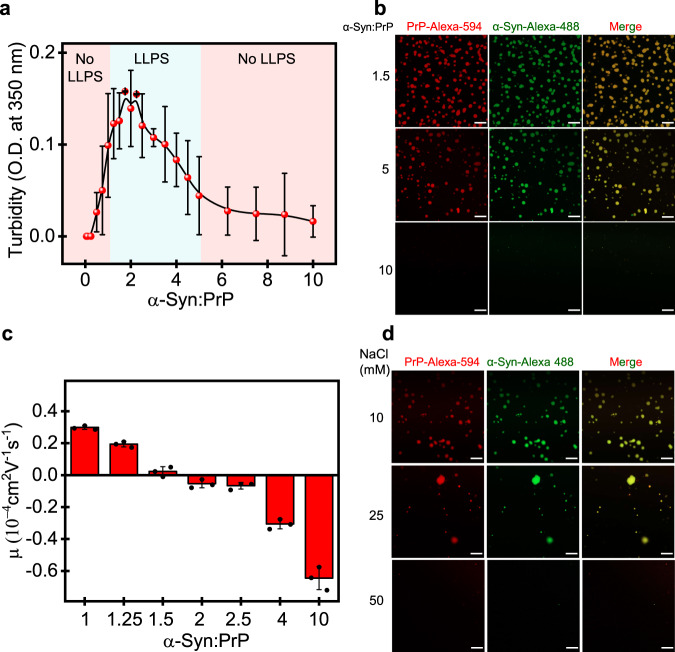
Charge neutralization drives heterotypic LLPS of α-Syn and PrP. **a** Solution turbidity plot at fixed PrP concentration (20 µM) as a function of increasing α-Syn concentrations showing reentrant phase behavior. The data represent mean ± s.d. for *n* = 4 independent experiments. The solid line is for eye guide only. **b** Confocal microscopy images of Alexa-594-labeled PrP (red) and Alexa-488-labeled α-Syn (green) at different stoichiometries as indicated. Scale bar: 10 µm. **c** Electrophoretic mobility (µ) measurements reveal charge inversion with the increase in the α-Syn:PrP ratio. The data represent mean ± s.d. for *n* = 3 independent experiments (corresponding data points are shown using black dot plots). **d** Confocal images of Alexa-594-labeled PrP (20 µM) and Alexa-488-labeled α-Syn (30 µM) droplets with increasing salt concentrations. Scale bar: 10 µm. The imaging was performed thrice with similar observations (**b**, **d**). Source data are provided as a Source Data file.

Charge neutrality is a primary condition for coacervate formation between counterionic electrolytes which occurs at a well-defined stoichiometry. To this end, we constructed phase diagrams as a function of the protein concentration using turbidity measurements. These measurements in the presence of an increasing concentration of α-Syn at a fixed PrP concentration revealed a typical reentrant phase behavior with three distinct regimes analogous to RNA-induced reentrant phase transitions (Fig. 2a)^56^. LLPS occurred within a narrow stoichiometry regime with the maximum LLPS at α-Syn:PrP molar ratio of ≈1.5–2. Any deviation from this stoichiometry resulted in the decrease in the LLPS propensity followed by the dissolution of these condensates, as was also confirmed using fluorescence imaging (Fig. 2b). The dissolution could be due to charge inversion in the presence of excess α-Syn. We next measured the electrophoretic mobility of solution which is indicative of the net surface charge on the peptides. The narrow LLPS regime exhibited an almost neutral net surface charge, whereas higher or lower α-Syn resulted in a charge inversion (Fig. 2c). In addition, a reverse titration experiment with increasing PrP concentration against a fixed α-Syn concentration displayed a similar phase behavior (Fig. S3a). To further support the role of electrostatic interactions, we carried out the phase separation assays as a function of increasing ionic strength (Fig. S3b). The addition of increasing amounts of salt resulted in the dissolution of these droplets (Fig. 2d). Higher protein concentrations were required to drive LLPS at higher ionic strengths. These results established the critical role of electrostatics in modulating the phase behavior for this counterionic polyelectrolyte mixture. Since electrostatic interactions appear to be the predominant LLPS driver, the next aspect was to investigate the role of temperature in governing the complex coacervation.

We next set out to study the thermo-responsive behavior associated with the complex coacervation of α-Syn and PrP. The phase behavior of a protein is encoded in its amino acid composition and is governed by a critical balance between protein–protein and protein-solvent interactions. The interaction network between protein and solvent is stabilized by contribution from the enthalpy and entropy of mixing, which drives the system to attain a minimum global free energy. The tendency of the protein to phase separate may increase or decrease with temperature giving rise to an LCST (lower critical solution temperature) or UCST (upper critical solution temperature) transitions, respectively^24^. Therefore, we next performed the LLPS assay for these coacervates as a function of temperature ranging from 4 to 42 °C. We observed an increase in phase separation propensity as a function of temperature indicating an LCST behavior, which is the signature of an entropy-driven system (Fig. S3c,d). The entropic gain associated with the release of counterions results in the LCST behavior^57,58^. Additionally, these droplets exhibited thermo-reversibility in multiple heating-cooling cycles (Fig. S3e). Taken together, these results suggest a predominant role of electrostatic interactions in driving this complex coacervation. We postulate that the positively charged N-terminal segment of PrP and negatively charged C-terminal domain of α-Syn participate in these intermolecular interactions. Therefore, we next set out to characterize the role of different domains of both PrP and α-Syn in the two-component phase transition.

### Domain-specific heterotypic interactions drive PrP-α-Syn condensation

**Fig. 3:**
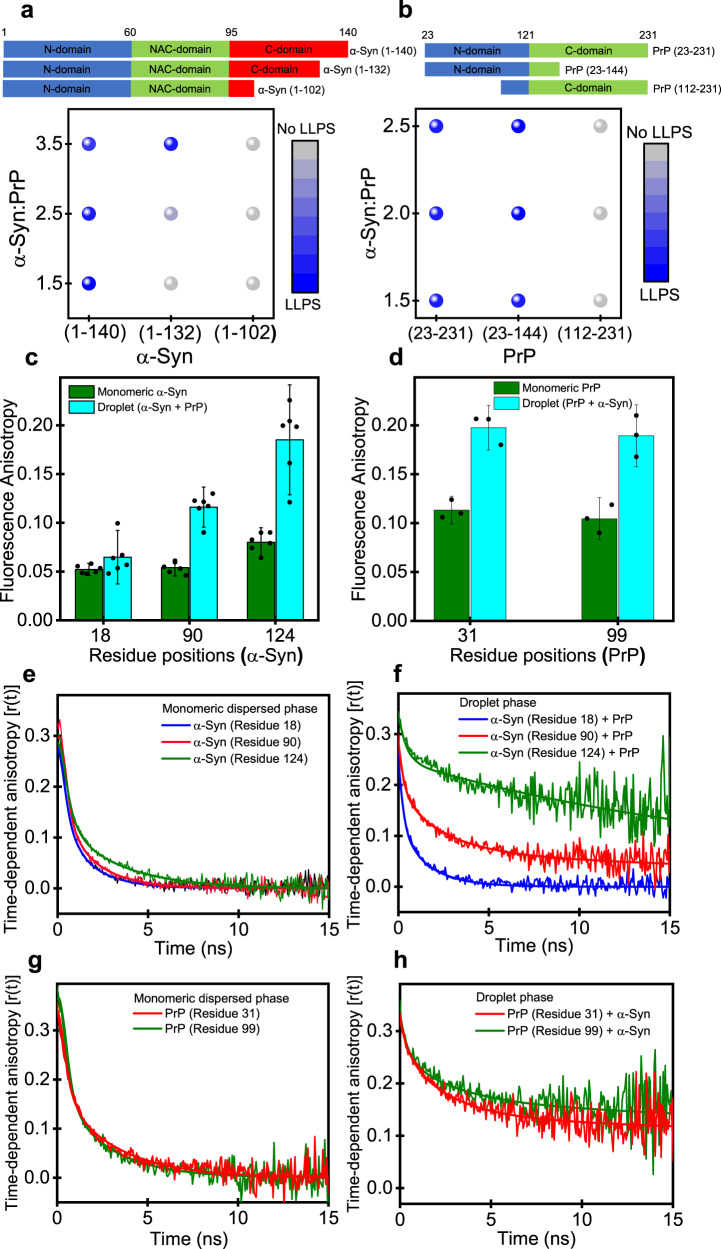
Domain-specific heterotypic interactions and the presence of electrostatic clusters within PrP-α-Syn condensates. **a** Schematic representation of different α-Syn constructs used. Phase diagram for different α-Syn constructs at fixed PrP concentration (20 µM) as a function of increasing α-Syn concentrations created from mean turbidity values. **b** Schematic representation of different PrP constructs used. Phase diagram for different PrP constructs (20 µM) as a function of increasing α-Syn concentrations created from mean turbidity values. **c** Steady-state fluorescence anisotropy of single-Cys α-Syn labeled at different positions using F5M in the mixed monomer (olive) and droplets (cyan). The data represent mean ± s.d. for *n* = 6 independent experiments (corresponding data points are shown as black dot plots). **d** Steady-state fluorescence anisotropy of PrP labeled at different positions using F5M in the monomer (olive) and droplets (cyan). The data represent mean ± s.d. for *n* = 3 independent experiments (corresponding data points are shown as black dot plots). **e**, **f** Time-resolved anisotropy decays of F5M-labeled α-Syn in dispersed α-Syn monomers and complex coacervate of PrP-α-Syn. **g**, **h** Time-resolved anisotropy decays of F5M-labeled PrP in dispersed PrP monomer and complex coacervate of PrP-α-Syn. The solid lines are fits obtained using the biexponential and triexponential decay analysis for monomers and droplets, respectively. See Methods, for details of picosecond time-resolved anisotropy decays measurements, analysis, and the estimation of R_h_. Source data are provided as a Source Data file.

In order to unveil the putative role of the negatively charged C-terminal domain of α-Syn in heterotypic LLPS, we created two naturally occurring C-terminally truncated variants of α-Syn, namely 133Stop (α-Syn 1–132) and 103Stop (α-Syn 1–102). These truncated variants are found in insoluble disease deposits associated with synucleinopathies^59^. The truncation lowers the net negative charge, the charge density, and NCPR. We hypothesized that shortening the acidic C-terminal tail would lower the LLPS propensity. In accordance with our expectation, our turbidity assay revealed a much lower propensity of α-Syn 1–132 to undergo heterotypic LLPS with PrP as compared to full-length α-Syn. α-Syn 1–102 containing an even shorter acidic tail did not exhibit LLPS with PrP (Fig. 3a, Fig. S4a, b). Our results revealed an imperative role of the C-terminal domain of α-Syn and demonstrated the critical role of charge density in modulating the two-component phase transition of α-Syn and PrP. Similarly, to verify the role of different PrP domains in promoting this heterotypic LLPS, we created naturally occurring truncations in PrP associated with prion diseases. We first questioned the role of the PrP globular domain in its complex coacervation with α-Syn. To this end, we created PrP 112–231 that retains the complete globular domain and a much shorter disordered N-terminal tail. PrP 112–231 did not exhibit LLPS even upon prolonged incubation with α-Syn under our experimental conditions (Fig. 3b, Fig. S4d). We next used a naturally occurring PrP deletion mutant PrP 23–144 (Y145Stop) which retains the disordered N-terminal tail of PrP and is devoid of the C-terminal folded domain^60^. Y145Stop exhibited a similar phase separation propensity as compared to full-length PrP in the presence of α-Syn, indicating the necessary and sufficient role of the N-terminal IDR of PrP in two-component LLPS with α-Syn (Fig. 3b, Fig. S4c).

Together, these results showcase the importance of electrostatic effects of two oppositely charged intrinsically disordered domains in complex coacervation. Our experimental observations are in accordance with bioinformatic predictions and demonstrate the critical role of PrP N-terminal and α-Syn C-terminal in dictating the two-component phase behavior. Previous studies with non-biological polyelectrolytes as well as IDPs have revealed the formation of primary nano-complexes as a preliminary step to the formation of complex coacervates^61,62^. These primary units comprising nano-complexes are believed to constitute the building blocks for these heterotypic coacervates. Once formed, these units can coalesce into a highly dynamic liquid-like dense phase that appears to be homogeneous at the mesoscopic length-scale but may contain some molecular/nanoscale order and dynamic heterogeneity. We hypothesized that region-specific electrostatic interactions could potentially induce short length-scale molecular or nanoscale ordering within the condensed phase. Therefore, in order to delineate the short-range order and dynamic heterogeneity at a high spatiotemporal resolution, we next utilized site-specific picosecond time-resolved fluorescence measurements that offer valuable residue-specific dynamic insights on the picosecond to nanosecond timescales.

### Presence of electrostatic nanoclusters within liquid droplets

To address the region-specific structural ordering, we utilized site-specific fluorescence anisotropy measurements that allow us to capture the local rotational flexibility. An increase in the steady-state fluorescence anisotropy at a residue location is attributed to an increase in order and a loss in conformational flexibility. In order to record the region-specific anisotropy, we took advantage of the fact that α-Syn is devoid of Cys and created single-Cys mutants at residues 18, 90, and 124 located at the N-terminal domain, NAC domain, and C-terminal domain, respectively, and labeled them using a thiol-active dye namely, fluorescein-5-maleimide (F5M). The steady-state fluorescence anisotropy of residue 18 located at the N-terminal domain of α-Syn did not exhibit any changes upon LLPS with PrP, whereas a C-terminal location of α-Syn at residue 124 showed a significant rise in the anisotropy upon phase separation with PrP indicating a critical contribution of the C-terminal domain of α-Syn in LLPS (Fig. 3c). The NAC-domain location α-Syn at residue 90 exhibited only a moderate increase in the anisotropy, which could hint at a possible role of hydrophobic residues in promoting such nanoscale ordering (Fig. 3c). To verify if the positively charged N-terminal disordered domain of PrP experiences a similar rotational constraint upon complex coacervation with α-Syn, we created single-Cys mutants at residues 31 and 99 of PrP and labeled them using F5M. A similar increase in the fluorescence anisotropy upon LLPS indicated a rotational hindrance for the N-terminal domain of PrP upon LLPS with α-Syn (Fig. 3d). The addition of salt disperses the droplets and results in lower anisotropy values similar to the dispersed phase indicating that the rise of anisotropy was indeed due to local ordering upon the complex coacervation (Fig. S5a). Together these results revealed that the highly basic N-terminal segment of PrP and the acidic C-terminal tail of α-Syn experience restricted rotational mobility within liquid droplets, corroborating our findings that domain-specific heterotypic electrostatic interactions drive the formation of two-component condensates of PrP and α-Syn. However, steady-state fluorescence measurements provide time-averaged information, and therefore, cannot distinguish between various modes of rotational dynamics of polypeptide chains. We next employed picosecond time-resolved fluorescence anisotropy measurements to disentangle distinct dynamical events on the nanosecond timescale.

Picosecond time-resolved fluorescence anisotropy decays yield fluorescence depolarization kinetics that permits us to capture the various modes of rotational relaxation of a polypeptide chain^63^. For instance, monomeric disordered conformers display a typical rapid depolarization that can be described by biexponential decay kinetics comprising a fast (sub-nanosecond) rotational correlation time representing the local fluorophore dynamics and a slower (nanosecond) rotational correlation time corresponding to the backbone dihedral rotations. For expanded IDPs, the slower correlation time represents a characteristic relaxation time (~1.4 ns) that arises due to short-range torsional fluctuations in the Φ-Ψ dihedral space^63,64^. As expected, in the monomeric dispersed phase, α-Syn exhibited such typical location-independent biexponential fluorescence depolarization kinetics for all the three locations (residues 18, 90, and 124) that is expected for a rapidly fluctuating expanded disordered state (Fig. 3e). Upon phase separation with PrP, the N-terminal location of α-Syn containing residue 18 retained the dynamic characteristics of a disordered chain, whereas, the C-terminal location at residue 124 exhibited significantly slower depolarization kinetics with a much slower rotational correlation time ~54 ns indicating local clustering around this region of α-Syn (Fig. 3f, Fig. S5b, c, Table S2). The depolarization kinetics also comprise a segmental chain dynamics component of ~4 ns. The NAC-domain position (residue 90) showed a moderate increase in the slower rotational correlation time as compared to the dispersed form (Fig. 3f). Similarly, the N-terminal segment of PrP that is highly dynamic in the monomeric dispersed form exhibited a significant dampening of rotational dynamics (Fig. 3g, h). In addition, we also used a single-Cys mutant near the C-terminal folded domain of PrP at residue 230 which exhibited a slower depolarization even in the monomeric dispersed phase due to the rotational tumbling of the globular domain. Although the C-terminal structured domain of PrP is not involved in intermolecular interactions as indicated by our truncation studies, a minor increase in the anisotropy and a bit slower rotational dynamics of the C-terminal domain upon LLPS could be due to the crowding effect within the droplets (Fig. S5d,e). These results are in accordance with our hypothesis that electrostatic interactions between the acidic C-terminal domain of α-Syn and basic N-terminal segment of PrP are the key drivers of α-Syn-PrP complex coacervation. Such domain-specific electrostatic interactions can yield partially and temporally ordered heterotypic oligomeric domains that can act as non-covalent crosslinks responsible for phase transitions. The hydrodynamic radius (R_h_) of such heterotypic domains estimated from the slower rotational correlation time (~54 ns) by using the well-known Stokes-Einstein relationship is ~4.3 nm (See Table S2)^65^. We would like to note that this is an approximation and the exact hydrodynamic size of the cluster will be dependent on the internal droplet viscosity. We, however, do not rule out the presence of other correlation chain fluctuations in the cluster. Nevertheless, our fluorescence depolarization kinetics indicated electrostatic clustering of C-terminal α-Syn and N-terminal PrP into relatively ordered nano-blobs acting as primary units of liquid droplets. The existence of such intermolecular clusters has recently been shown in tau condensates^66^. We posit that these clusters that are detected on the nanosecond timescale can undergo breakings and makings of transient crosslinks on a much slower timescale resulting in a liquid-like behavior on a longer length and timescales as observed by FRAP. On a slower timescale of tens of seconds in FRAP measurements, both proteins diffuse giving rise to a rapid and complete recovery. Such spatiotemporal regulations might be relevant for aberrant phase transitions and the modulation of the phase behavior by RNA and other biomolecules.

### RNA participates in a competitive multicomponent coacervation

**Fig. 4:**
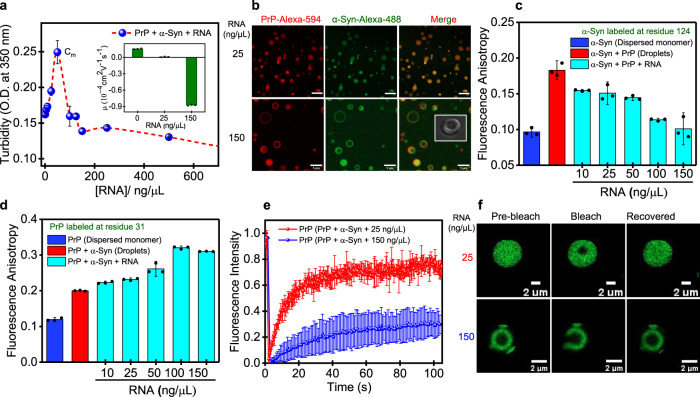
RNA participates in a competitive multicomponent coacervation. **a** Solution turbidity plot as a function of increasing polyU RNA at a fixed α-Syn:PrP ratio showing a reentrant phase behavior. The inset shows charge inversion at different RNA concentrations. The data represent mean ± s.d. for *n* = 3 independent experiments (corresponding data points are shown as black dot plots) **b** Confocal fluorescence images for different regions of the phase diagram with RNA concentrations as indicated. Before the maximum (C_m_), the ternary complex exhibits miscibility, whereas, beyond the maximum (C_m_), the droplets start dispersing by transitioning into multiphasic, vesicle-like, hollow condensates. The imaging was performed thrice with similar observations. Top panel: PrP (20 µM) + α-Syn (30 µM) + RNA (25 ng/µL). Scale bar: 10 µm. Bottom panel: PrP (60 µM) + α-Syn (90 µM) + RNA (150 ng/µL). Scale bar: 5 µm. The inset shows a DIC image for a single hollow condensate (Scale bar: 10 µm). See Supplementary Movie 2 for hollow condensates. **c** Steady-state fluorescence anisotropy for F5M-labeled α-Syn at residue 124 indicating its displacement from the condensates with increasing RNA concentrations. The data represent mean ± s.d. for *n* = 3 independent experiments (corresponding data points are shown as black dot plots). **d** Steady-state fluorescence anisotropy for F5M-labeled PrP residue 31 indicating an increase in the order within hollow condensates with the increase in the RNA concentration. The data represent mean ± s.d. for *n* = 3 independent experiments (corresponding data points are shown as black dot plots). **e** FRAP kinetics of multiple droplets (~1% Alexa-488-labeled protein) for PrP at different RNA concentrations (25 ng/µL: blue; 150 ng/µL: red). The data represent mean ± s.d. for *n* = 3 independent experiments. **f** Fluorescence images of droplets during FRAP measurements. Source data are provided as a Source Data file.

Most phase-separated condensates are enriched in RNA and harbor proteins with RNA-binding domains^4^. Competing protein–protein and protein–RNA interactions underlie the compositional specificity of these cellular condensates in a context-dependent manner^67,68^. Therefore, we next set out to characterize the role of RNA in regulating this phase transition. PrP contains an RNA-binding domain and is known to interact with nucleic acids^69^. We first tested the phase separation propensity of PrP and α-Syn separately in the presence of RNA. PrP exhibited LLPS in the presence of RNA and displayed reentrant phase behavior as also observed previously for other prion constructs (Fig. S6a, b)^60,70^. However, α-Syn did not undergo phase separation upon the addition of RNA, as reported previously (Fig. S6c)^38^. We next studied the behavior of preformed PrP-α-Syn complex coacervates in the presence of RNA. Turbidity measurements complemented with microscopic observations revealed a reentrant phase transition of these heterotypic condensates in the presence of RNA under our experimental conditions (Fig. 4a upper panel). Low RNA/protein ratios promoted phase separation resulting in the formation of ternary droplets that appear to have a uniform distribution (Fig. 4b lower panel). However, beyond a threshold RNA concentration, it resulted in multiphasic droplets with vesicle-like morphology (Fig. 4a, b, S6d). Previous studies have also shown the formation of these vesicle-like hollow condensates in the presence of RNA^56,71^. We hypothesize that the repulsion due to the charge inversion together with a competition between α-Syn and RNA for binding sites on PrP resulted in the formation of these multiphasic condensates beyond a certain RNA concentration. Our electrophoretic mobility measurements supported the charge inversion phenomenon due to a high net negative surface charge at higher RNA concentrations (Fig. 4a inset). The addition of RNA to the preformed PrP-α-Syn coacervates resulted in the displacement of α-Syn from these droplets, indicating a stronger affinity of RNA towards PrP. The displacement of α-Syn by RNA was also confirmed using steady-state fluorescence anisotropy. F5M-labeled residue 124 of α-Syn revealed a sharp increase in the fluorescence anisotropy upon LLPS; however, the addition of increasing concentration of RNA progressively disrupted its interaction with PrP, as revealed by the gradual decrease in the fluorescence anisotropy (Fig. 4c). In addition, SDS-PAGE of sedimented droplets in the presence of RNA indicated a much lower amount of α-Syn, suggesting its weak partitioning into these RNA-rich condensates (Fig. S6e). To test if these hollow condensates exhibit spatial ordering, we measured fluorescence anisotropy for PrP, which is highly enriched within these vesicle-like condensates. The F5M-labeled residue 31 of PrP exhibited a sharp increase in the anisotropy upon addition of RNA, indicating the presence of molecular ordering within these multiphase condensates (Fig. 4d). Additionally, PrP exhibited much slower FRAP recovery within these hollow condensates corroborating our fluorescence anisotropy measurements (Fig. 4e, f). Such a molecular ordering might be generic to nucleoprotein vesicles, as has also been shown previously^71^. LLPS is highly sensitive to fluctuations in concentrations of the components inside the cellular milieu. The addition of RNA to the preformed PrP-α-Syn heterotypic droplets results in the switching of coacervate morphology and composition. We speculate that such competing interactions might be present in the cellular environment depending upon the subcellular locations. For instance, exosomes with high RNA concentrations might not be conducive for PrP-α-Syn heterotypic interactions. Taken together, our results indicate an RNA-induced tuning of the compositional specificity of these condensates in a context-dependent manner. It is interesting to note that such interactions can provide spatiotemporal regulations; however, the high enrichment of biomolecules within these condensates makes them a susceptible site for aberrant phase transitions and pathological aggregation under stress conditions. Therefore, we next set out to elucidate the effect of α-Syn-PrP complex coacervation on the aggregation propensity of these heterotypic condensates.

### Synergistic heterotypic interactions promote liquid-to-solid amyloid transition

**Fig. 5:**
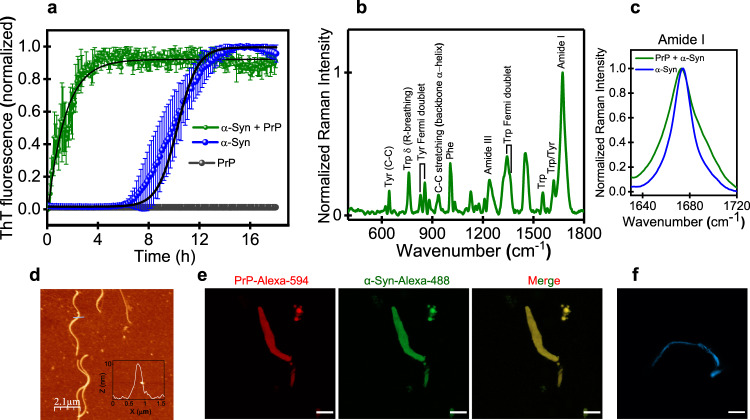
Synergistic heterotypic interactions promote a liquid-to-solid amyloid transition. **a** ThT kinetics for de novo aggregation α-Syn (30 µM) and PrP (20 µM) (separately) and LLPS-mediated aggregation via a liquid-to-solid transition of complex coacervates of α-Syn and PrP completely bypassing the lag phase. The data represent mean ± s.d. for *n* = 3 independent experiments. The black solid lines are fits for isodesmic (olive colored plot) and nucleation-dependent polymerization kinetics (blue colored plot). Source data are provided as a Source Data file. **b** Vibrational Raman spectra of PrP-α-Syn aggregates indicating their heterotypic nature. **c** Amide I is shown for comparison between PrP-α-Syn heterotypic aggregates formed via LLPS and α-Syn homotypic aggregates formed via de novo aggregation. **d** AFM image of LLPS-mediated heterotypic aggregates showing the presence of typical amyloid fibrils. The inset shows the height profile (~10 nm). **e** Two-color confocal fluorescence images showing colocalization of α-Syn and PrP within these heterotypic aggregates. **f** Confocal fluorescence image of a ThT-positive fibril. Scale bar: 10 µm. The imaging was performed twice with similar observations (**d**, **f**).

LLPS-mediated liquid-to-solid phase transitions into pathological aggregates have been observed for various other neuronal IDPs/IDRs such as FUS, TDP43, prion, and so forth^32,72^. PrP and α-Syn pathologies have been together implicated in prion diseases. For instance, extensive α-Syn immunoreactive deposits accumulate in the brains of CJD patients^42^. Also, colocalization of α-Syn and PrP aggregates has been observed in early cytoplasmic inclusions bodies^73^. Therefore, we next set out to determine if α-Syn-PrP heterotypic interactions within these complex coacervates can promote aggregation and amyloid formation. We first monitored the aggregation kinetics of these droplets using a well-known amyloid marker Thioflavin-T (ThT). An increase in ThT fluorescence was observed after 40 h of incubation indicating the presence of ThT-positive aggregates indicating a slow liquid-to-solid transition of these condensates (Fig. S7a). We also performed time-dependent CD spectroscopy to gain insights into the structural attributes of these aggregates which showed the presence of β-rich aggregates after 40 h (Fig. S7b). Transmission electron microscopy also indicated the presence of amyloid-like aggregates along with some amorphous aggregates indicating a solid-to-liquid transition (Fig. S7c). To further mimic the crowded cellular environment as well as to accelerate the liquid-to-solid transition, we next monitored the kinetics of conversion using ThT under stirring conditions that speed up the aggregation presumably due to a faster nucleation process. α-Syn alone exhibited typical nucleation-dependent polymerization kinetics with a lag phase of ~6 h, as expected, whereas PrP alone did not form ThT-positive aggregates under our experimental condition (Fig. 5a). However, heterotypic α-Syn-PrP coacervates upon incubation under a stirring condition rapidly aggregated into amyloids via isodesmic kinetics by completely bypassing the long lag phase (Fig. 5a). In contrast, aggregation at various ratios under non-LLPS conditions did not eliminate the lag phase indicating the critical role of heterotypic condensates in accelerating the amyloid transition via LLPS-mediated pathway (Fig. S8a).

We next structurally characterized these LLPS-mediated heterotypic aggregates using vibrational Raman spectroscopy that allowed us to elucidate the different secondary structural elements present within these aggregates. The amide I vibrational band that originates primarily due to the C=O stretching of the backbone appeared at 1675 cm^−1^, which is the hallmark of a hydrogen-bonded cross-β amyloid architecture (Fig. 5b)^74^. The full-width at half maximum (FWHM) of amide I and III for α-Syn-PrP heterotypic amyloids were considerably broader (31 ± 2 cm^−1^ for amide I) compared to homotypic α-Syn amyloids (20 ± 1 cm^−1^ for amide I) (Fig. 5c, Fig. S8b, c). A broader amide I band indicated the contribution from the globular α-helical domain of PrP which, at least in part, is possibly retained in α-Syn-PrP heterotypic amyloids (Fig. 5c). Our CD data also indicated the presence of both helical and β-rich conformers in these amyloids, corroborating our Raman results (Fig. S8d). Additionally, tryptophan residues present in the N-terminal of PrP experience decreased polarity as evident from its small Raman blue-shift to 883 cm^−1^ indicating that the N-terminal part of PrP might be sequestered in the core of α-Syn-PrP amyloids. Further, to visualize these heterotypic amyloid aggregates, we performed atomic force microscopy (AFM) which revealed the presence of typical nanoscopic amyloid fibrils with a height of ~10 nm (Fig. 5d and inset). Additionally, the two-color high-resolution Airy scan confocal fluorescence imaging revealed colocalization of PrP and α-Syn within these amyloid fibrils (Fig. 5e). Also, LLPS-mediated aggregates demonstrated a solid-like behavior as indicated by no FRAP recovery (Fig. S8e,f). Together, these results reveal synergistic effects of α-Syn and PrP within liquid-like complex coacervates allowing their conformational sequestration and rapid conversion into highly ordered, solid-like ThT-active amyloid fibrils (Fig. 5f).

## Discussion

**Fig. 6:**
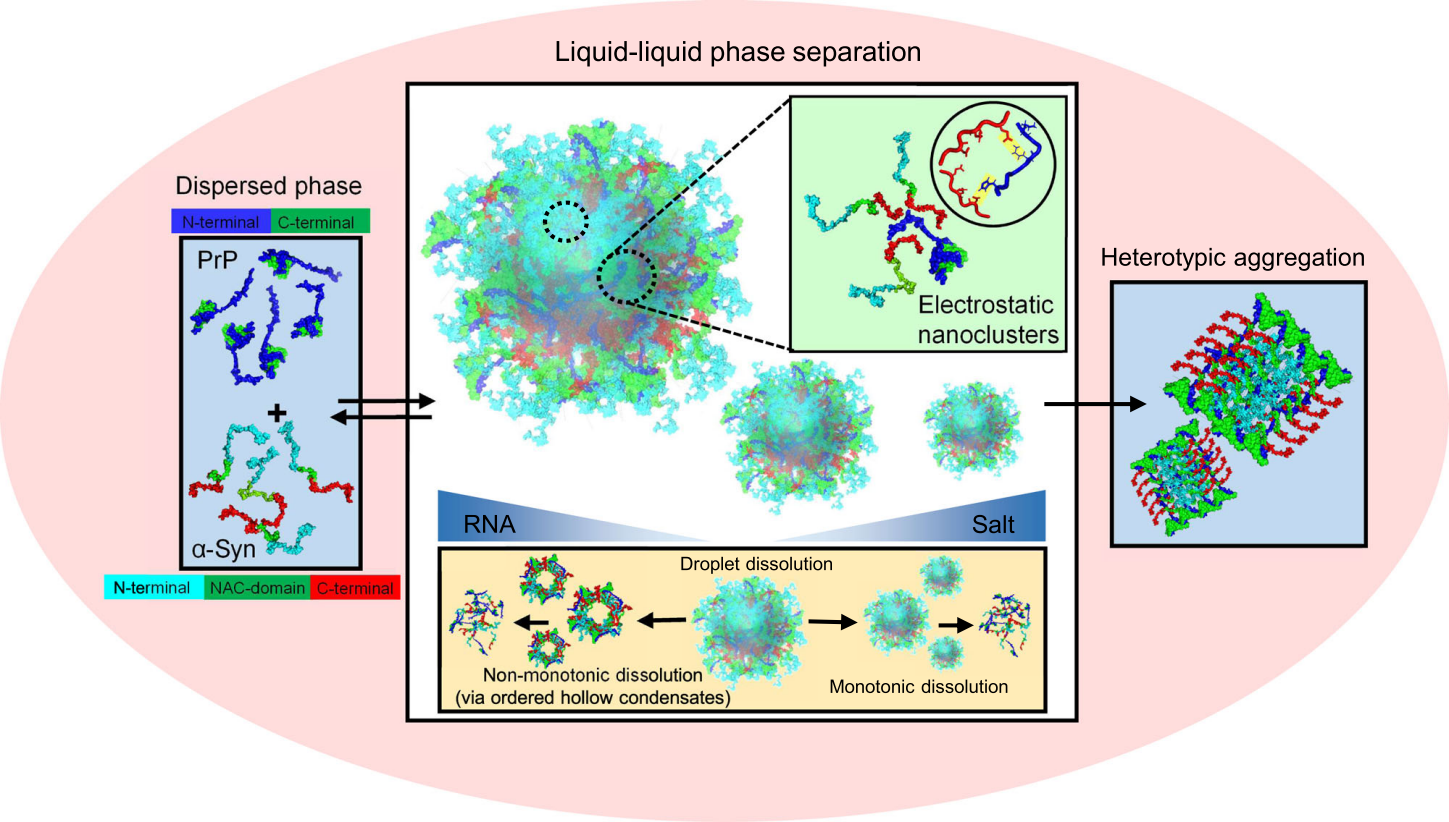
A schematic of PrP-α-Syn-RNA multicomponent condensates. Complex coacervation of PrP and α-Syn drives the formation of partially ordered transient electrostatic nanoclusters. The addition of salt results in a monotonic condensate dissolution, whereas, the addition of RNA results in a non-monotonic dissolution via multiphasic hollow condensates. PrP-α-Syn condensates undergo a liquid-to-solid transition into heterotypic amyloids.

In this work, we showed that domain-specific electrostatic interactions between PrP and α-Syn at a narrow stoichiometry regime result in the formation of highly dynamic liquid-like droplets with a mobile internal organization (Fig. 6). The charge neutralization drives the formation of these condensates, whereas, the charge inversion promotes their dispersion into a homogeneous solution akin to RNA-induced reentrant behavior. The entropic gain associated with counterion release upon LLPS allows these coacervates to display an LCST phase behavior. We demonstrated the critical role of different domains in promoting LLPS by using deletion mutations that revealed that the N-terminal disordered segment of PrP and the C-terminal domain of α-Syn are the principal drivers of two-component LLPS. Our site-specific picosecond time-resolved fluorescence anisotropy measurements revealed the formation of relatively ordered, transient, electrostatic nanoclusters that are stable on the nanosecond timescale. These clusters can act as oligomeric subunits connected via physical crosslinks within the condensed phase (Fig. 6). Our results also underscore the importance of timescales of breaking-and-making of non-covalent interactions in governing the hierarchical architecture and internal material property at different length scales. On the nanosecond timescale and molecular-to-nano length-scale, there can be a considerable structural and dynamical heterogeneity, whereas, on a much slower (seconds) timescale and mesoscopic length-scale, these assemblies display typical liquid-like characteristics. Such characteristics might be generic for a wide variety of multicomponent condensates.

In summary, our results demonstrate that phase separation can potentially offer a mechanism of modulation of the interactions between PrP and α-Syn. Both PrP and α-Syn have been shown to be localized in lipid rafts^75,76^. Although PrP is a well-folded GPI-anchored cell-surface protein, its intrinsically disordered N-terminal segment retains a high degree of flexibility and multivalency that enables promiscuous interactions with multiple binding partners. IDR-enabled LLPS is emerging as one of the potential mechanisms for assembling cell-surface receptors to facilitate signal transduction^8,77^. Given the presence of both PrP and α-Syn within the lipid rafts, we speculate that the receptor clustering in lipid rafts might be one of the potential mechanisms to promote complex coacervation of PrP and α-Syn in the cellular context. Our findings also demonstrate the buffering role of RNA in their interactions. The addition of RNA to these preformed condensates weakens the α-Syn-PrP interaction and disrupts the formation of the ordered domains. Lower RNA concentrations yielded ternary droplets with a uniform distribution, whereas higher RNA concentrations resulted in the formation of ordered, multiphasic, vesicle-like condensates with charge-driven hierarchal architecture (Fig. 6). Such modulations in heterotypic interactions might potentially act as a framework to understand competitive protein–protein and protein–RNA interactions within the cellular milieu^67^.

The interaction of α-Syn with the N-terminal IDR of PrP is reminiscent of the recruitment of amyloid-β oligomers by PrP involved in synaptic impairment hinting at a plausible role of heterotypic α-Syn-PrP interactions in neurotoxicity^78^. Recent studies have also provided evidence for the role of PrP as a membrane-surface receptor in the context of α-Syn oligomers and fibrils^43–45,79^. The interaction elicits neurotoxic signaling pathways through metabotropic glutamate receptor 5, Fyn kinase, and N-methyl D-aspartate receptors resulting in synaptic dysfunction. Additionally, the presence of extensive α-Syn deposits has been linked to unique CJD cases with longer disease courses^45,80,81^. Colocalization of α-Syn and PrP aggregates has been observed in early cytoplasmic inclusions^73^. Our findings showcase the synergistic effect of PrP and α-Syn interactions in complex biomolecular condensates that can act as reaction crucibles to catalyze aberrant liquid-to-solid phase transitions into early heterotypic amyloids. Our study also highlights the pertinent role of LLPS in driving interactions between PrP and α-Syn, which under normal cellular conditions may be reversible; however, cellular stress can promote their aberrant phase transitions. Interestingly, such heterotypic interactions might potentially be involved in blocking prion propagation as shown previously^45,82,83^. Furthermore, the heterotypic buffering in the presence of RNA can offer an alternate pathway in regulating their phase behavior and might be important in suppressing their potentially toxic effects. It is important to note that both PrP and α-Syn are transported via extracellular vesicles and exosomes, which are highly enriched in RNA and perhaps can act as potential sites for such multiphasic interactions^84–86^. The interplay of these critical molecular events can have broad implications in physiologically relevant receptor-mediated signaling as well as in disease-associated aberrant phase transitions.

## Methods

### Bioinformatic analyses of prion protein and α-synuclein using various prediction tools

Classification of Intrinsically Disordered Ensemble Regions (CIDER)^51^ (http://pappulab.wustl.edu/CIDER/analysis) was used to predict the charge distribution and net charge per residue (NCPR) of human PrP and α-Syn. IUPred2a (https://iupred2a.elte.hu/)^87^ was used to predict the intrinsic disorder. FuzPred/FuzDrop (http://protdyn-fuzpred.org/)^52^ and catGRANULE (http://s.tartaglialab.com)^53^ were used to predict the LLPS propensity of both PrP and α-Syn. All these data were plotted using Origin.

### Site-directed mutagenesis, protein expression, and purification

Recombinant full-length human PrP (PrP 23–231) plasmid cloned in vector pRSET-B was transformed in BL21(DE3)pLysS. PrP 23–144 (Y145Stop), PrP 112–231, and single cysteine variants of full-length PrP (W31C, W99C, and S230C) were created using site-directed mutagenesis^60,88^. Recombinant full-length human α-Syn (1–140) plasmid cloned in vector pT7.7 was transformed in BL21(DE3)pLysS^89^. α-Syn (1–102) (N103Stop) and α-Syn (1–132) (Y133Stop) were created using the full-length α-Syn plasmid. The primers used for α-Syn mutations are listed in Table S1. Single cysteine variants of α-Syn (A18C, A90C, and A124C) were created using site-directed mutagenesis^89^. Recombinant prion protein constructs were overexpressed and purified using nickel-NTA affinity chromatography^60,88^. The purified proteins were refolded using the PD10 column in 14 mM MES buffer, pH 6.8. Recombinant α-Syn constructs except 103Stop were purified using the anion-exchange chromatography as described previously^89^. α-Syn 103Stop was purified using cation-exchange chromatography. The purified proteins were dialyzed overnight for buffer exchange (14 mM MES buffer, pH 6.8). The purity of all the proteins was confirmed by SDS-PAGE analysis. Protein concentrations were estimated using ε_280 nm_ = 56,590 M^−1^cm^−1^ for PrP(23–231), ε_280 nm_ = 43,670 M^−1^cm^−1^ for Y145Stop, ε_280 nm_ = 14,200 M^−1^cm^−1^ for PrP (112–231), ε_278 nm_ = 5600 M^−1^cm^−1^ for wt α-Syn, ε_278 nm_ = 3840 M^−1^cm^−1^ for α-Syn 133Stop, ε_278 nm_ = 1280 M^−1^cm^−1^ for α-Syn 103Stop. All the experiments were performed using freshly purified proteins.

### Fluorescence labeling

Labeling of single cysteine variants were performed in denaturation buffer at pH 7.5 using thiol-active fluorescent dyes namely, fluorescein-5-maleimide (F5M), AlexaFluor-488-C5-maleimide (Alexa-488), AlexaFluor-594-C5-maleimide (Alexa-594), and 5-((2-((iodoacetyl)amino)ethyl)amino)naphthalene-1-sulfonic acid (IAEDANS). Excess free dye was removed using a PD10 desalting column. Labeled protein concentrations were estimated using molar extinction coefficients of the dyes [ε_495 nm_ = 68,000 M^−1^cm^−1^, for F5M; ε_493 nm_ = 72,000 M^−1^cm^−1^, for Alexa-488; ε_588 nm_ = 96,000 M^−1^cm^−1^ for Alexa-594 and ε_337 nm_ = 6100 M^−1^cm^−1^ for IAEDANS].

### Dynamic light scattering (DLS) and electrophoretic mobility measurements

Hydrodynamic radii were estimated using the DLS instrument (Malvern Zetasizer). All the buffers were filtered through 0.02 µm filters before measurements. Monomeric PrP (50 µM), α-Syn (50 µM) and droplets (PrP + α-Syn; 20 µM + 30 µM) were used for the measurements. Electrophoretic mobility measurements were estimated at 25 °C using the DLS instrument using the M3-PALS (Phase Analysis Light Scattering) method.

### Phase separation assays

Phase separation was induced by mixing α-Syn, PrP, and RNA in the desired stoichiometries. The turbidity of the phase-separated samples for PrP-α-Syn-RNA complex (25 °C), PrP-RNA coacervates (25 °C), and PrP-wild-type with α-Syn/103Stop/133Stop (37 °C) were measured at 350 nm on Genova Life Science spectrophotometer (ver.1.51.4). The mean and the standard error were obtained from at least three independent sets of samples. The turbidity of the phase-separated samples for α-Syn-wild-type with PrP (23–144)/ PrP (112–231) (37 °C) and PrP-α-Syn at different temperatures were estimated by taking absorbance at 350 nm on a Multiskan Go (Thermo scientific) plate reader using 96-well NUNC optical bottom plates. For temperature-dependent turbidity assays, the LLPS-induced solution was incubated for 5 min at respective temperatures to minimize any discrepancy due to temperature fluctuation. The sample volume used for these measurements was 150 μL, and raw turbidity data are plotted with background subtraction. For most of the experiments, the PrP concentration was fixed to 20 μM, and the α-Syn concentration was fixed at 30 μM, pH 6.8 unless otherwise mentioned.

The protein and buffer solutions were separately equilibrated at the given temperature for 2 min before mixing. All the samples were prepared independently and the measurements were made immediately after mixing within 30 seconds.

### Sedimentation assays

For sedimentation assays, complex coacervates (100 µL) of PrP (20 µM) and α-Syn (30 µM) were formed and were incubated for 5 min. They were then centrifuged at 25,000 × *g*, 25 °C to separate dense phase and light phase. The supernatant was carefully removed, and the pellet (dense phase) obtained after centrifugation was resuspended in 8 M urea (10 µL). The samples were run on an SDS-PAGE (15%) and were visualized using the Coomassie blue staining. The saturation concentration (C_sat_) was estimated by comparing the supernatant intensity with a known concentration intensity using ImageJ software. Hollow condensates of α-Syn-PrP in the presence of RNA (150 ng/µL) were also processed similarly. Single-stranded polyU RNA with a molecular weight of 800–1000 kDa was obtained from Sigma Aldrich. RNA concentration was estimated using a Genova Life Science spectrophotometer (ver.1.51.4).

### Confocal microscopy

All the imaging experiments were performed at room temperature on ZEISS LSM 980 Elyra 7 super-resolution microscope equipped with a high-resolution monochrome cooled AxioCamMRm Rev. 3 FireWire(D) camera, using a ×63 oil-immersion objective (numerical aperture 1.4). For visualizing droplets of PrP-α-Syn and PrP-α-Syn-RNA complexes, 1% of proteins was doped with the labeled proteins, and the samples were placed in Labtek chambered coverglass. Imaging using coverslips also yielded similar results. Alexa-488-labeled protein was imaged using a 488 nm laser diode (11.9 mW), and Alexa-594-labeled protein was imaged using a 590 nm excitation source. The ThT-positive fibrils were imaged using a 402 nm excitation source. The images were obtained at a resolution of 1840 × 1840 pixels at 16 bit depth. Images were processed and analyzed using ImageJ (NIH, Bethesda, USA). Concentrations in the dense phase and the light phase were estimated as follows^90^. Calibration plots were generated from fluorescence intensities of the dispersed phase of Alexa-488-labeled α-Syn and Alexa-594-labeled PrP at different concentrations. Using similar acquisition settings, the confocal images were acquired for droplets formed using Alexa-488-labeled PrP, and Alexa-594-labeled α-Syn using 0.1% labeled protein. Thresholding was performed to eliminate the background signal from the droplet intensity. Droplets were then analyzed using the ImageJ software to obtain the total area and mean fluorescence intensity for each droplet. The standard calibration plot of Alexa-488-labeled PrP and Alexa-594-labeled α-Syn was used to interpolate the approximate estimated concentration of PrP and α-Syn, respectively. The C_sat_ was estimated using a similar method using 2% labeled protein and was verified using SDS-PAGE analysis as described above. All the imaging experiments were performed at room temperature.

### Fluorescence recovery after photobleaching (FRAP)

FRAP experiments were performed on ZEISS LSM 980 Elyra 7 super-resolution microscope equipped with a high-resolution monochrome cooled AxioCamMRm Rev. 3 FireWire(D) camera, using a ×63 oil-immersion objective (Numerical aperture 1.4). Alexa-488-labeled α-Syn and PrP (~1%) were used for FRAP experiments (measurements were performed for at least three independent samples). A region of interest (ROI) with a radius of 0.5 μm was bleached using a 488 nm laser for PrP-α-Syn hetero-protein coacervates. The recovery of the bleached spots was recorded using ZEN Pro 2011(ZEISS) software provided with the instrument. Time-dependent FRAP was performed by taking aliquots from droplets reaction at mentioned time points. The fluorescence recovery curves were background corrected, normalized, and plotted using Origin.

### Circular dichroism spectroscopy (CD)

The far-UV CD experiments for time-dependent CD experiments and aggregates were recorded on a Biologic MOS500 spectrometer at room temperature in a 1 mm pathlength quartz cuvette. For time-dependent CD measurements under quiescent conditions, the samples were centrifuged at 25,000 × *g*, 37 °C, and the CD spectra were acquired after resuspending the pellets in 20 mM phosphate buffer, pH 7.5. The aggregates of PrP-α-Syn (20 µM + 30 µM) and α-Syn after 18 h (under stirring conditions) were centrifuged at 25,000 × *g*, 37 °C to separate the monomeric population. All the spectra were averaged over three scans and were blank subtracted, which were then processed and plotted using Origin.

### Aggregation kinetics

The thioflavin T (ThT) aggregation kinetics were performed using NUNC 96-well plate on POLARstar Omega Plate Reader Spectrophotometer (BMG LABTECH, Germany) at 37 °C. The reaction mixtures (150 µL) containing a glass bead were subjected to stirring conditions, 600 rpm, with protein concentrations mentioned in the respective plots. The final concentration of ThT in the reaction mixture was 20 μM. For bulk Raman measurements, similar aggregation reactions were set up without ThT in the reaction mixture. The kinetic traces were plotted using Origin.

### Steady-state fluorescence anisotropy

The steady-state fluorescence experiments were performed on a FluoroMax-4 spectrofluorometer (Horiba Jobin Yvon, NJ, USA) using a 1 mm pathlength quartz cuvette. Fluorescence measurements were performed for α-Syn-PrP condensates at 37 °C and α-Syn-PrP-RNA condensates at 25 °C. The concentrations of PrP and α-Syn were fixed at 20 µM and 30 µM, respectively. For recording fluorescence, F5M-labeled PrP and α-Syn (200 nM of labeled protein mixed with the unlabeled protein) were used. The samples were excited at 485 nm and the emission spectra were collected in the range between 510 nm and 600 nm. For recording fluorescence of IAEDANS-labeled single-Cys 124 of α-Syn (15 µM of labeled protein was mixed with the wild-type protein), the samples were excited at 375 nm, and the emission spectra were collected in the range between 400 nm and 600 nm. Single point readings and emission spectra were recorded for ThT fluorescence. The samples were incubated at 37 °C under quiescent conditions. Readings were taken at the indicated time points and the samples were excited at 440 nm. Twenty micromolar of ThT was used for the experiments. The steady-state fluorescence anisotropy data of labeled proteins were recorded at emission maxima (measurements were performed for at least three independent samples). The steady-state fluorescence anisotropy (*r*_ss_) was estimated using the following relationship.

1 $${r}_{{ss}}=\frac{{I}_{\parallel }-{{GI}}_{\perp }}{{I}_{\parallel }+2G{I}_{\perp }}$$

where *I*_∥_ and *I*_⊥_ are the parallel and perpendicular fluorescence intensities, respectively, and the measured intensities were corrected using the G-factor.

### Picosecond time-resolved fluorescence anisotropy measurements

Time-resolved fluorescence anisotropy decay measurements were performed using a time-correlated single-photon counting (TCSPC) set up (Horiba Jobin Yvon, NJ). The samples were excited using 485 nm and 375 nm NanoLED picosecond laser diodes for F5M and IAEDANS-labeled proteins, respectively. The instrument response function (IRF) was obtained by using a dilute solution of colloidal silica (Ludox) and the full-width half maxima (FWHM) was estimated to be ~265 ps. All the measurements were performed at 37 °C. To record anisotropy decay profiles, the emission wavelength was set at the respective emission maxima, with a bandpass of 8 nm. The fluorescence intensities were collected at 0° (*I*_II_) and 90° (*I*_⊥_) with respect to the geometric orientation of the excitation polarizer. The perpendicular fluorescence intensity decays were corrected using the *G*-factor obtained from free dyes in water. Measurements were performed for at least three independent samples. The anisotropy decays were analyzed by global fitting of *I*_II_ and *I*_⊥_ using the following equations:

2 $$\,{I}_{\parallel }\left(t\right)=1/3I\left(t\right)\left[1+2r\left(t\right)\right]$$

3 $$\,{I}_{\perp }\left(t\right)=1/3I\left(t\right)\left[1-r\left(t\right)\right]$$

where *I* represent the time-dependent fluorescence intensity collected at the magic angle (54.7°). The time-resolved depolarization kinetics is described using a typical biexponential decay function, which defines fast (*ϕ*_1_) and slow (*ϕ*_2_) rotational correlation times arising due to local motion of the fluorophore and segmental mobility of IDP, respectively^63^.

4 $$r\left(t\right)={r}_{0}\,\left[{\beta }_{1}{e}^{\left(-\frac{t}{{\phi }_{1}}\right)}+\,{\beta }_{2}{e}^{\left(-\frac{t}{{\phi }_{2}}\right)}\right]$$

where *r*_0_ represents the intrinsic time zero or the fundamental anisotropy of the attached fluorophore. *β*_1_ and *β*_2_ represent the amplitudes associated with fast and slow rotational correlation time, respectively. The goodness of fit was evaluated by reduced *χ*^2^ values, the randomness of the residuals, and autocorrelation functions^63^.

For dispersed monomers and droplets (α-Syn residue 18), the anisotropy decays were fitted using a biexponential equation. However, for droplets (α-Syn residue 90, 124; PrP residue 31 and 99), time-resolved anisotropy decays could only be described by a triexponential decay model with an additional slower correlation time (*ϕ*_3_) as shown below.

5 $$r\left(t\right)={r}_{0}\,\left[{\beta }_{1}{e}^{\left(-\frac{t}{{\phi }_{1}}\right)}+{\beta }_{2}{e}^{\left(-\frac{t}{{\phi }_{2}}\right)}+\,{\beta }_{3}{e}^{\left(-\frac{t}{{\phi }_{3}}\right)}\right]$$

In order to get a better estimate of the slow correlation time (*ϕ*_3_) corresponding to the electrostatic clusters, a longer lifetime label (IAEDANS with a mean lifetime of ~12 ns) was used for the measurements. The *ϕ*_3_ was found to be 54 ± 6 ns (For recovered parameters, see Table S2). This value was used for estimating the approximate hydrodynamic radii (*R*_h_) of the nanoclusters. The *R*_h_ was estimated using the Stokes-Einstein equation as follows.

6 $${\phi }_{3}=\,\frac{\eta V}{{K}_{B}T}$$

where *η* is the viscosity of the medium, *V* is the volume of the rotating unit (*V* = ^4^/_3_π*R*_h_^3^), *k*_*B*_ is the Boltzmann constant, and *T* is the temperature. The robustness of the recovered correlation time (*ϕ*_3_) was also assessed by using both free and forced fits.

### Raman spectroscopy

Raman spectra for heterotypic PrP-α-Syn aggregates and homotypic α-Syn aggregates were recorded on an inVia laser Raman microscope (Renishaw, UK). The aggregates were centrifuged at 25,000 × g, and the obtained pellets were resuspended in 5 µL of 20 mM phosphate buffer, pH 7.5. The sample volume of 5 µL was deposited and dried onto a glass slide covered with an aluminum sheet. The sample was focused using a ×100 objective lens (Nikon, Japan), and a 785 nm 500 mW NIR laser with a 50% laser power and exposure time of 10 s was used for excitation. The Rayleigh scattering was filtered by using an edge filter of 785 nm. The Raman scattering was collected and dispersed using a 1200 lines/mm diffraction grating and detected using an air-cooled CCD detector. Inbuilt Wire 3.4 software was used for data acquisition. All the data were averaged over 20 scans. Baseline correction and smoothening of the acquired spectra were performed using Wire 3.4 and the spectra were plotted using Origin.

### Transmission electron microscopy (TEM)

LLPS-induced PrP-α-Syn samples were kept for incubation for ~36 h. The sample was then centrifuged at 25,000 × *g* at 37 °C, and the obtained pellet was resuspended in 20 mM phosphate buffer, pH 7.5. Three microliters of the resuspended solution was spotted on a 300-mesh carbon-coated electron microscopy grid and was incubated for 5 min. The grid was stained with 5 µL of uranyl acetate (1% w/v) following which the excess stain was removed and the sample was then allowed to dry overnight. The TEM images were acquired using Jeol JEM F-200.

### Atomic force microscopy (AFM)

AFM images of PrP-α-Syn co-aggregates were acquired on Innova atomic force microscope (Bruker) operating in tapping mode. For sample preparation, 10 μL of the aliquots were taken from the reaction mixture and were deposited onto the freshly cleaved, Milli-Q water-washed muscovite mica (Grade V-4 mica from SPI, PA). The samples were incubated for 5 min at room temperature and were washed twice with 100 μL of filtered Milli-Q water. The samples were further dried under a gentle stream of nitrogen before AFM imaging. NanoDrive (v8.03) software was used for the data acquisition and the acquired images were processed using WSxM 5.0D 8.1 software^91^. The height profiles were obtained from WSxM software and were plotted using Origin.

### Reporting summary

Further information on research design is available in the Nature Research Reporting Summary linked to this article.

## Supplementary information

Supplementary Information

Description of additional Supplementary File

Supplementary Movie 1

Supplementary Movie 2

Reporting Summary

## Data Availability

The data are available within the Article, Supplementary Information, or Source Data file. PED (Protein Ensemble Database) and PDB (Protein Data Bank) IDs used in this study are available on PED and PDB web servers. PED ID: PED00024e001 PDB ID: 2LSB. Source data are provided with this paper.

## Source data

Source Data
