# Supplementary material for: Spatiotemporal modulations in heterotypic condensates of prion and α-synuclein control phase transitions and amyloid conversion
Source: Nat Commun. 2022 Mar 3;13:1154. doi: 10.1038/s41467-022-28797-5 (PMC8894376; doi:10.1038/s41467-022-28797-5)
Supplement: Supplementary file 1 — Supplementary Information [file 41467_2022_28797_MOESM1_ESM.pdf]

Supplementary Information

Spatiotemporal Modulations in Heterotypic Condensates of Prion and α-Synuclein Control Phase Transitions and Amyloid Conversion

Aishwarya Agarwal,[1,2] Lisha Arora,[1,3] Sandeep K. Rai,[1,3] Anamika Avni,[1,3] and Samrat Mukhopadhyay[1,2,3]*

[1]Centre for Protein Science, Design and Engineering, [2]Department of Biological Sciences, and [3]Department of Chemical Sciences, Indian Institute of Science Education and Research (IISER) Mohali, Punjab, India.

*Corresponding author: Email: mukhopadhyay@iisermohali.ac.in

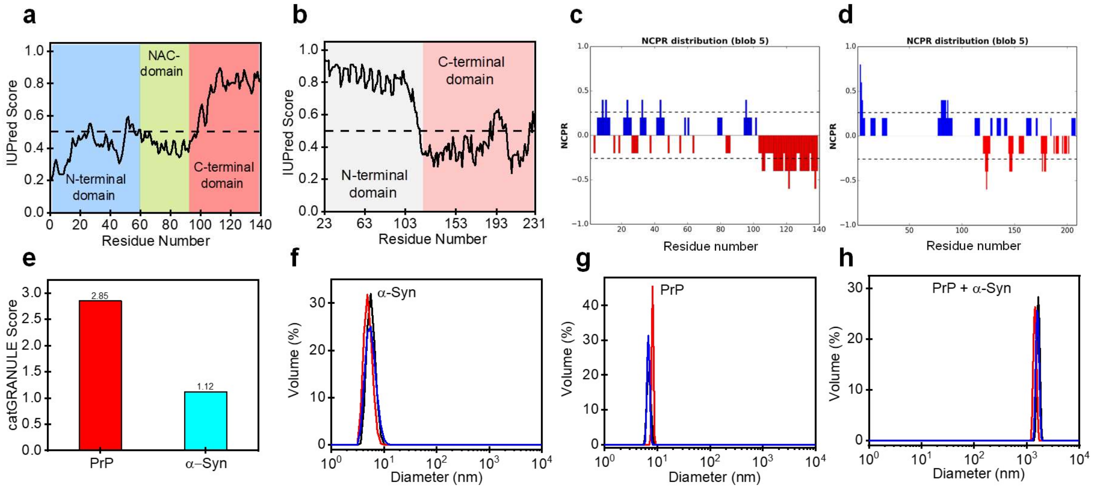

Fig. S1 Prediction of intrinsic disorder using IUPred for **a** α-Syn and **b** full-length PrP (PrP 23-231). Net charge prediction tool CIDER indicating positively charged residues (blue) and negatively charged residues (red) for **c** α-Syn and **d** PrP (23-231). **e** Prediction of phase separation propensity of PrP and α-Syn using catGRANULE (red: PrP, cyan: α-Syn). Particle size distribution using dynamic light scattering for **f** α-Syn monomer (50 μM) **g** PrP monomer (50 μM) and **h** PrP-α-Syn droplets (20 μM + 30 μM). The three different lines (red, black, and blue) correspond to three independent replicates of each sample.

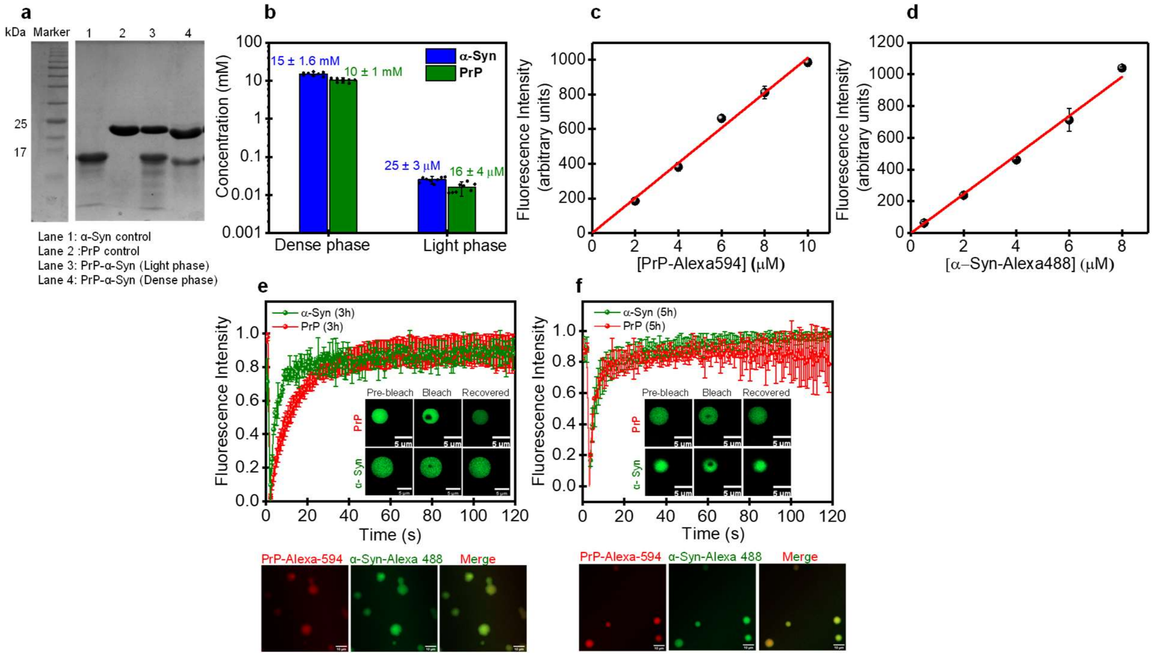

Fig. S2 **a** SDS-PAGE for PrP-α-Syn droplets after sedimentation assay. The experiment was performed twice with similar observations. **b** Dense phase and light phase concentrations estimation for α-Syn (blue) and PrP (olive) within droplets using fluorescence intensity calibration. The data represents mean ± s.d. for n = 8 droplets. Corresponding data points are shown as black dot plots. **c,d** The fluorescence intensity calibration plot using different concentrations of Alexa-488-labeled α-Syn and Alexa-594-labeled PrP. **e,f** FRAP kinetics of multiple droplets (~1% Alexa-488-labeled protein) for PrP (red) and α-Syn (olive) after 3 hours (e) and 5 hours (f). The data represents mean ± s.d. for n = 3 independent experiments. Inset shows fluorescence images of droplets during FRAP measurements. PrP and α-Syn concentrations were 20 μM and 30 μM, respectively. Confocal images for indicated time points are also shown. Scale bar: 10 μm. See source data file for source data.

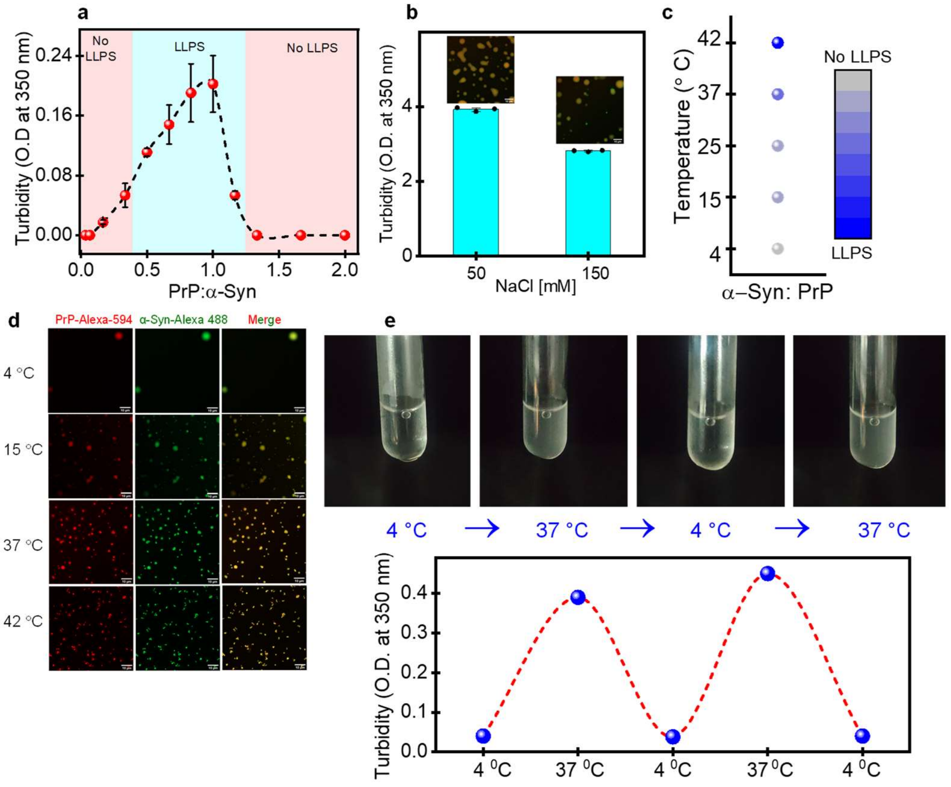

Fig. S3 a Solution turbidity plot at a fixed α-Syn concentration as a function of increasing PrP concentrations showing reentrant behavior. The data represents mean ± s.d. for n = 3 independent experiments. The dotted line is shown as an eye guide. **b** Solution turbidity plot at fixed higher PrP and α-Syn concentrations (PrP 60 μM and α-Syn 90 μM) at different salt concentration. The data represents mean ± s.d. for n = 3 independent experiments (corresponding data points are shown as black dot plots). The inset shows the respective confocal image indicating the presence of liquid droplets. **c** Phase diagram for α-Syn:PrP (1.5) as a function of increasing temperature constructed from mean turbidity values. **d** Confocal images of Alexa-594-labeled PrP (20 μM) and Alexa-488-labeled α-Syn (30 μM) droplets at different temperatures (Scale bar: 10 μm). The imaging experiments were performed at room temperature within 1 min of transferring. The experiments were performed twice with similar observations. **e** The thermo-reversibility cycle of heterotypic condensates of α-Syn (45 μM) and PrP (30 μM) between 4 °C and 37 °C shown by both photography and by turbidity measurements (the dotted line is shown as an eye guide). Photographs are of the same solution in the same test tube after incubation at different temperatures during the cycle. One of the photographs of the same phase-separated sample at pH 6.8 at 37 °C has also been shown in Fig. 1g of the main paper for comparison with homogeneous non-phase-separated solutions.

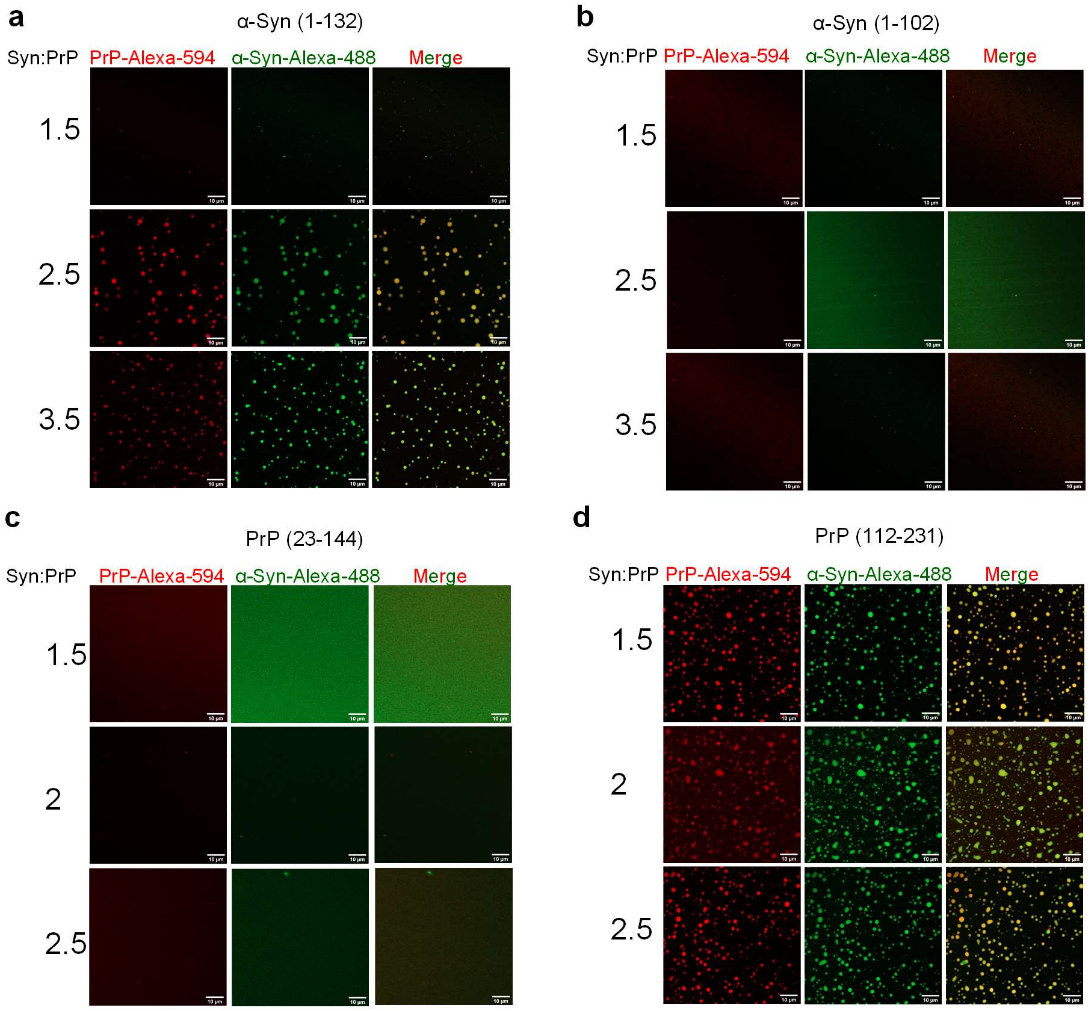

Fig. S4 a,b Confocal fluorescence images of Alexa-594-labeled PrP with different constructs of Alexa-488-labeled α-Syn. PrP concentration was fixed at 20 μM. Scale bar: 10 μm. **c,d** Confocal fluorescence images of Alexa-488-labeled α-Syn with different constructs of Alexa-594-labeled PrP. PrP concentration was fixed at 20 μM. Scale bar: 10 μm. All the experiments were performed twice with similar observation.

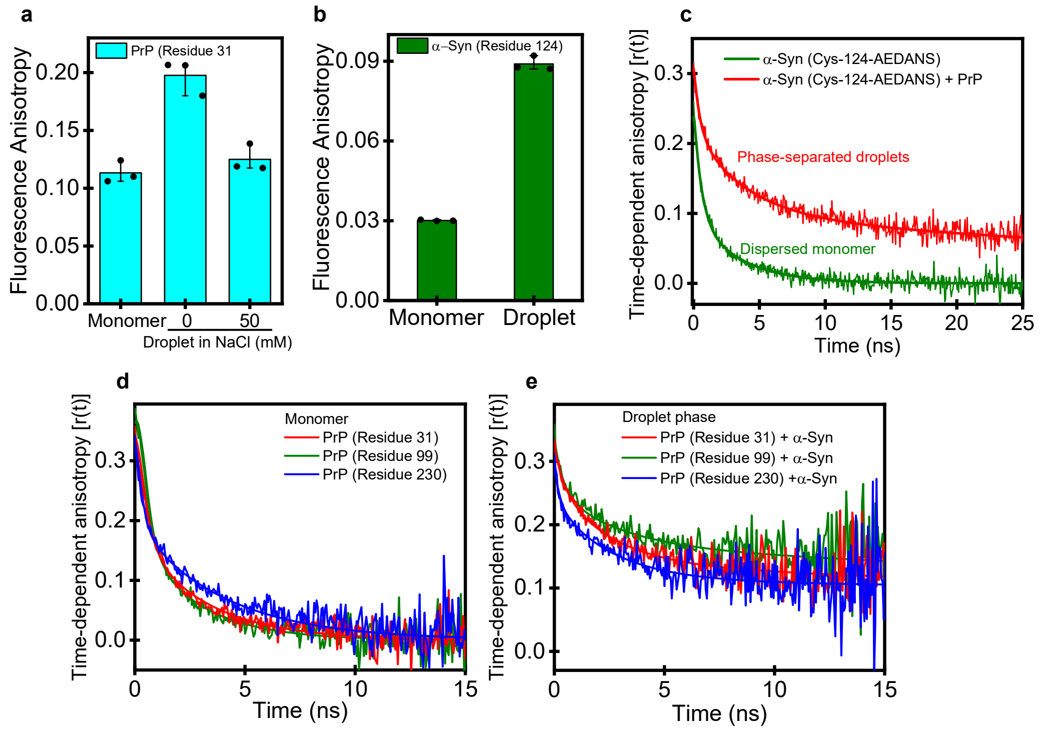

Fig. S5 **a** Steady-state fluorescence anisotropy for F5M-labeled PrP residue 31 indicating droplet dissolution in the presence of NaCl. The data represents mean ± s.d. for n = 3 independent experiments (corresponding data points are shown as black dot plots). **b** Steady-state fluorescence anisotropy for IAEDANS-labeled α-Syn residue 124. The data represents mean ± s.d. for n = 3 independent experiments (corresponding data points are shown as black dot plots). **c** Time-resolved anisotropy decay of IAEDANS-labeled α-Syn residue 124 in dispersed monomer (olive) and droplets (red). **d,e** Time-resolved anisotropy decays of F5M-labeled PrP at different residue locations in dispersed monomers and complex coacervates of PrP and α-Syn. Same plots (red and green traces) have been shown in Fig. 3g,h and are used here for comparison with residue 230 (blue). See source data file for source data.

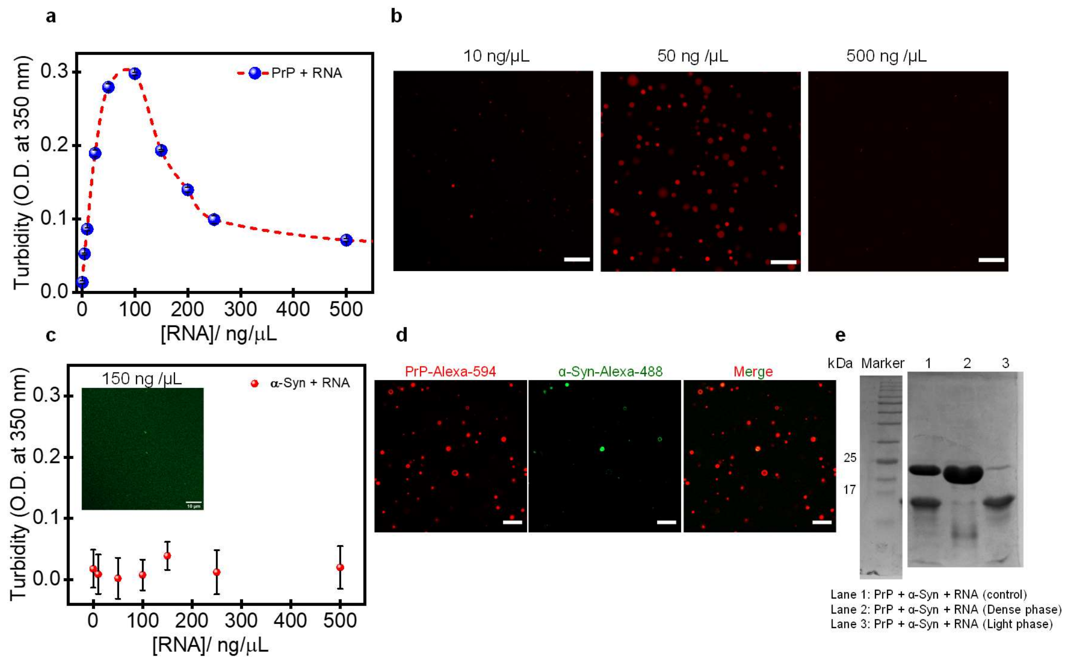

Fig. S6 a Solution turbidity plot for PrP as a function of polyU RNA concentration. The data represents mean ± s.d. for n = 3 independent experiments. The dotted line is shown as an eye guide. **b** Confocal images for Alexa-594-labeled PrP at different RNA concentrations as indicated. Scale bar: 10 µm. **c** Solution turbidity plot for α-Syn (30 µM) as a function of polyU RNA concentration. The data represents mean ± s.d. for n = 3 independent experiments. Inset shows a confocal image for Alexa-488-labeled α-Syn in presence of polyU RNA (150 ng/µL). **d** Confocal images of Alexa-594-labeled PrP (20 µM) and Alexa-488-labeled α-Syn (30 µM) droplets in the presence of RNA (150 ng/µL). Scale bar: 10 µm. The images were obtained at resolution of 512 x 512 pixels at 8 bit depth. The experiments were performed three times with similar observations (b, d) **e** SDS-PAGE analysis of PrP-α-Syn droplets in the presence of RNA (150 ng/ µL) using sedimentation assay. The experiments were performed twice with similar observations. See source data file for source data.

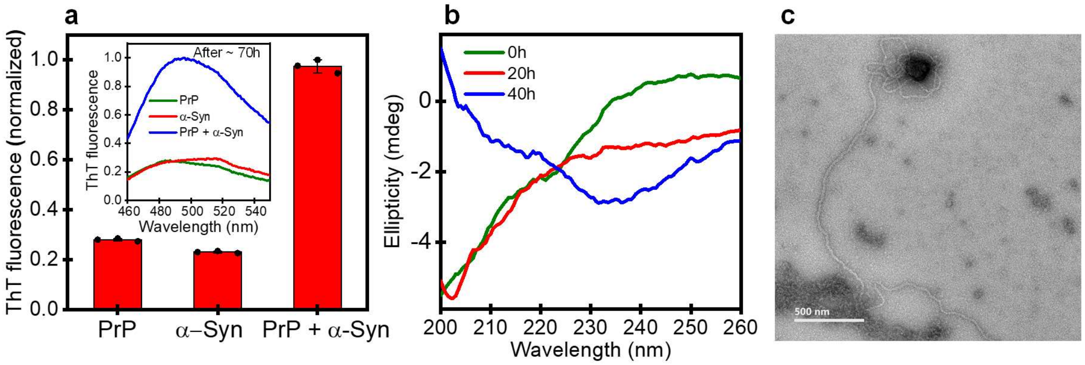

Fig. S7 a ThT fluorescence intensity of PrP (20 μM), α-Syn (30 μM) and PrP (20 μM) + α-Syn 30 μM) after ~ 70 h of incubation under the quiescent condition showing LLPS-mediated maturation and aggregation of PrP-α-Syn heterotypic condensates. ThT-positive aggregates are formed after ~ 40 h of incubation under the quiescent condition. The inset shows the ThT fluorescence spectra. The data represents mean ± s.d. for n = 3 independent experiments (corresponding data points are shown as black dot plots). **b** Time-dependent far-UV CD spectrum under quiescent conditions indicating a transition to β-rich amyloid-like aggregates. **c** TEM image of aggregates formed after ~ 40 h of incubation under the quiescent condition indicating the presence of amyloid-like fibrils with some amorphous aggregates. The experiment was performed twice with similar observations. See source data file for source data.

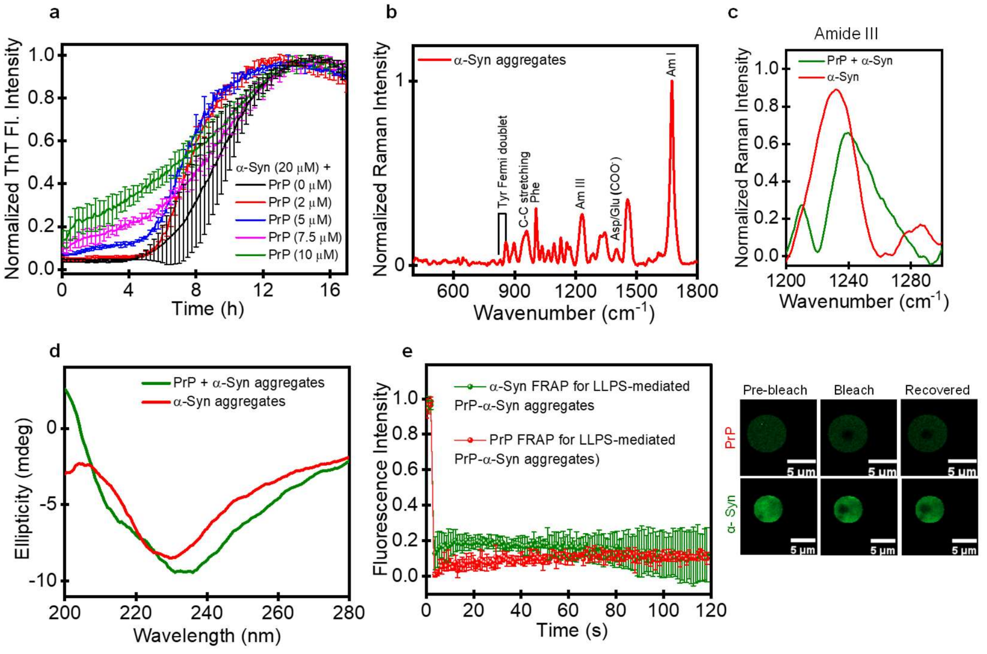

Fig. S8 a ThT aggregation kinetics for α-Syn in the presence of increasing PrP concentrations indicating shortening of lag-phase. The data represent mean ± s.d. for n = 3 independent experiments. **b** Vibrational Raman spectra of α-Syn aggregates. **c** The amide III region is shown for comparison between PrP-α-Syn co-aggregates and α-Syn homotypic aggregates. **d** Far-UV CD spectrum indicating β-rich structures for α-Syn homotypic aggregates whereas a mixture of α-helical and β-rich structure for PrP-α-Syn co-aggregates. **e** FRAP kinetics of later aggregates (1% Alexa-488-labeled protein) for PrP (red) and α-Syn (olive) indicating a liquid-to-solid transition (fluorescence images of circular regions of interest during FRAP measurements are shown). The data represent mean ± s.d. for n = 3 independent experiments. See source data file for source data.

Table S1: Primer sequences.

Construct	Primer	Sequence 5'-3'
α-Syn (103 Stop)	Forward	GGACCAGTTGGGCAAGAATTAAGAAGGATGCCCACAGG
	Reverse	GTGGGCATCCTTCTTAATTCTTGCCCAACTGGTCC
α-Syn (133 Stop)	Forward	CCTTCTGAGGAAGGGTATTAAGACTATGAACCTGAAGCC
	Reverse	GGCTTCAGGTTCATAGTCTTAATACCCTTCCTCAGAAGGC
α-Syn (A18C)	Forward	GGGAGTTGTGGCTTGTGCTGAGAAAACCAAACAGGG
	Reverse	CCCTGTTTGGTTTTCTCAGCACAAGCCACAACTCCC
α-Syn (A90C)	Forward	GCAGGGAGCATTGCATGTGCCACTGGCTTTGTC
	Reverse	GACAAAGCCAGTGGCACATGCAATGCTCCCTGC
α-Syn (A124C)	Forward	GCCTGTGGATCCTGACAATGAGTGCTATGAAATGCC
	Reverse	GGCATTTCATAGCACTCATTGTCAGGATCCACAGGC
PrP (23-144)	Forward	CGGCAGTGACTAGGAGGACCGTTAC
	Reverse	GTAACGGTCCTCCTAGTCACTGCCG
PrP (112-231)	Forward	AAAAGCTAGCATGGCTGGTGCTGCAGCAGC
	Reverse	GCTTCGAATTCTCAGGACGATCCTC
PrP W31C	Forward	GAAGCCTGGAGGATGTAACACTGGG
	Reverse	CCCAGTGTTACATCCTCCAGGCTTC
PrP W99C	Forward	CCCACAGTCAGTGTAACAAGCCGAG
	Reverse	CTCGGCTTGTTACACTGACTGTGGG
PrP S230C	Forward	CCAGAGAGGATGCTCCTGAGAATTCG
	Reverse	CGAATTCTCAGGAGCATCTCCTCTGG

Table S2: Recovered parameters from the time-resolved fluorescence anisotropy decay analysis for α-Syn labeled by IAEDANS at Cys 124 in dispersed phase and droplets.

α-Syn		ϕ_1 (β_1)	ϕ_2 (β_2)	ϕ_3 (β_3)
**Residue 124**	Dispersed monomer	0.51 ± 0.07 ns (0.64 ± 0.03)	3.38 ± 0.39 ns (0.36 ± 0.03)	-
	Droplets	0.43 ± 0.05 ns (0.34 ± 0.03)	3.77 ± 0.42 ns (0.36 ± 0.01)	54.26 ± 6.02 ns (0.31 ± 0.02)
