# Supplementary material for: Spatiotemporal modulations in heterotypic condensates of prion and α-synuclein control phase transitions and amyloid conversion
Source: Nat Commun. 2022 Mar 3;13:1154. doi: 10.1038/s41467-022-28797-5 (PMC8894376; doi:10.1038/s41467-022-28797-5)
Supplement: Supplementary file 2 — Description of additional Supplementary File [file 41467_2022_28797_MOESM2_ESM.pdf]

Descriptions for Additional Supplementary Material Files

Supplementary Movie 1 : Fusion events liquid droplets formed via complex coacervation of PrP (30 μM) and α-Syn (40 μM).

Supplementary Movie 2 : Hollow condensates of PrP (60 μM) and α-Syn (90 μM) in the presence of RNA (150 ng/μl).
